# The Role of Artificial Intelligence in Theranostics

**DOI:** 10.2967/jnmt.125.270251

**Published:** 2025-12

**Authors:** Geoffrey M. Currie, Eric Rohren

**Affiliations:** 1 Charles Sturt University, Wagga Wagga, New South Wales, Australia; and; 2 Baylor College of Medicine, Houston, Texas

**Keywords:** theranostics, radionuclide therapy, artificial intelligence, deep learning, generative AI

## Abstract

The recent reinvigoration of theranostics comes with advances in computing technology, radiochemistry, and instrumentation that synergize with developments in artificial intelligence (AI). There is a wide array of applications of AI in nuclear medicine that have translational benefits to theranostics, including attenuation correction, artifact and noise reduction, enhanced workflow, and lesion characterization, and segmentation and quantitation, among many others. For theranostics, there are potentially significant applications that could move closer to precision medicine. Perhaps the most important application is predictive dosimetry from diagnostic images to optimize therapeutic dose. There are also valuable benefits from AI-augmented radioligand design and development, preclinical imaging, and practice sustainability. Generative AI has also emerged as a powerful tool to support decision-making, information dissemination, and medical image analysis. There are, however, several ongoing challenges that must be considered pertaining to the development and application of AI tools in theranostics.

The second coming of artificial intelligence (AI) in nuclear medicine has emerged at the same time as the revitalization of theranostics. There is a long history of the application of the principles of AI and theranostics to nuclear medicine practice. This reinvigoration comes with advances in computing technology, radiochemistry, and instrumentation that synergize with the developments in AI and targeted therapies. Indeed, advances in molecular imaging and precision outcomes of radionuclide therapies have demanded optimization in data processing, radiomic feature extraction, radiation dosimetry and workflow that AI augmentation provides the logical solution.

AI is a broad term that includes machine learning (ML) as a subdomain (Fig. 1). ML includes artificial neural networks and a range of other algorithms. Among the artificial neural networks, both discriminatory and generative models have emerged, with applications in nuclear medicine favoring deep (i.e., consisting of many layers) neural networks that are referred to as deep learning (DL). The architecture and operation of the various AI algorithms have been described previously (1–4). A wide range of applications of AI in nuclear medicine could also benefit theranostics and have been discussed in detail previously (1,3,5). In brief, these include automation of labor-intensive image segmentation and quantitation across multiple modalities and multiple imaging times (therapy images and diagnostic images) (6,7), more accurate and reproducible delineation of tumor volumes for radiomic feature extraction (8), application of AI-driven radiomic feature extraction to evaluate tumor response to therapy and predict the response to therapy (6–11), differentiation between disease grades (12–16), and predicting recurrence and outcomes and crafting accurate nomograms (6,7).

**FIGURE 1.**
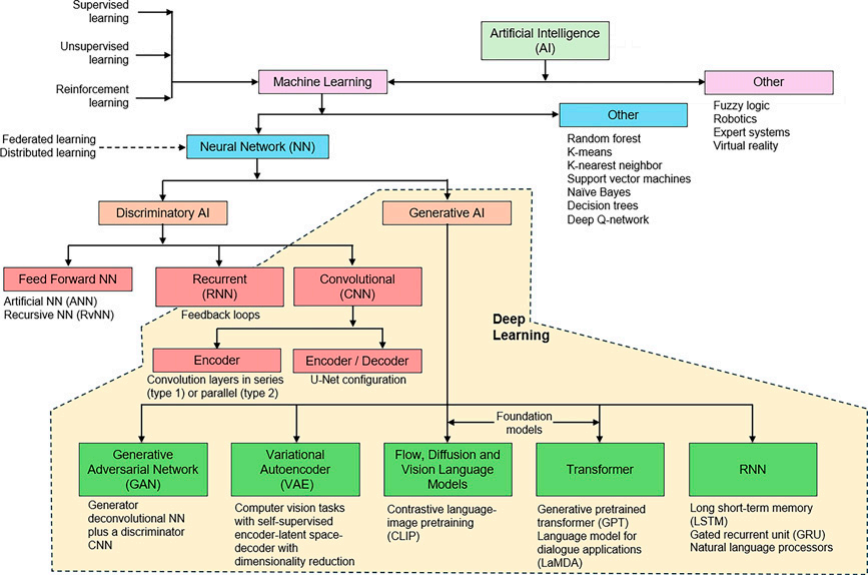
Classification of AI, ML, DL, and generative AI.

There are also several important applications with potential implications in theranostics that warrant more detailed discussion. AI-augmented pseudo-CT attenuation-correction maps have been used to reduce the radiation dose to patients from CT or PET/MRI, where no CT attenuation map is produced (17–21). In PET/MRI, convolutional neural network (CNN) approaches have shown superiority (2% variation from CT attenuation map) over current approaches (e.g., maximum likelihood reconstruction of attenuation, Dixon method) (18,21,22). In theranostics, CNN-based attenuation correction could be particularly important in reducing the CT-based radiation dose during serial imaging and improving posttherapy ^177^Lu serial SPECT images with accurate attenuation correction without CT. For example, serial posttherapy ^177^Lu SPECT may include a real CT scan performed at a single time point for the additional benefits of accurate anatomic localization and replace the remaining SPECT time points with pseudo-CT images for attenuation correction.

DL-generated ^177^Lu SPECT/CT and PET/CT reconstruction using encoder–decoder CNNs can reduce artifact and noise in reconstructed images compared with both filtered background projection and iterative approaches (23–25). For theranostics, whether diagnostic PET/CT and SPECT/CT or posttherapy (e.g., ^177^Lu) serial SPECT images, improved reconstruction with noise reduction and less artifact will improve the accuracy of quantitation, radiomic features, and precision. SPECT imaging with ^177^Lu can also have CNN-based U-Net partial-volume–effect correction, which improves the accuracy of quantitation (26).

Radiation dose reduction in CT and denoising PET and SPECT images enhance segmentation (27–30). Dose reduction of PET radiopharmaceuticals has been a focus of AI augmentation but, given the cumulative doses of theranostics, is less a priority than precision theranostics and improved patient outcomes. An important consideration when using denoising algorithms for low-dose or low-count imaging studies in theranostics is that, although the image produced is enhanced, the data remain the same. As a consequence, the low- count statistical noise will undermine the certainty of quantitation and radiomic features. In turn, this could undermine precision theranostics and patient outcomes.

There are, however, innovative roles for AI, specifically for the enhancement of theranostics and a broader discussion of these will follow. Lesion characterization and radiomic feature extraction, including textual features and deep radiomic features, offer a powerful tool to move toward precision theranostics (7,10,31,32). AI-augmented radiation dosimetry from serial posttherapy images enhances reactive dosimetry (9). AI-augmented predictive dosimetry from diagnostic images is needed to realize precision theranostics and individualize therapeutic doses (9,33,34). There are also valuable benefits from AI-augmented radioligand design and development, including reduced cost and development time (35). AI-based digital twins can also be used to reduce the cost and development time of preclinical phases of radioligand development and to improve sustainability (33,35,36). Indeed, AI can be used to improve sustainability across the theranostics ecosystem and to address diversity (37,38). Federated learning is a tool that allows big data for CNN training in theranostics without breaching privacy or confidentiality, addressing ethical issues and sustainability (39–42). Generative pretrained transformers (GPTs) and large language models (LLMs) can be adopted to support decision-making through extraction of textual information from clinical guidelines (43,44) and generate reports (7). Vision language models (VLMs) have an emerging role in medical image analysis (45–47).

## RADIOMIC FEATURE EXTRACTION

**FIGURE 2.**
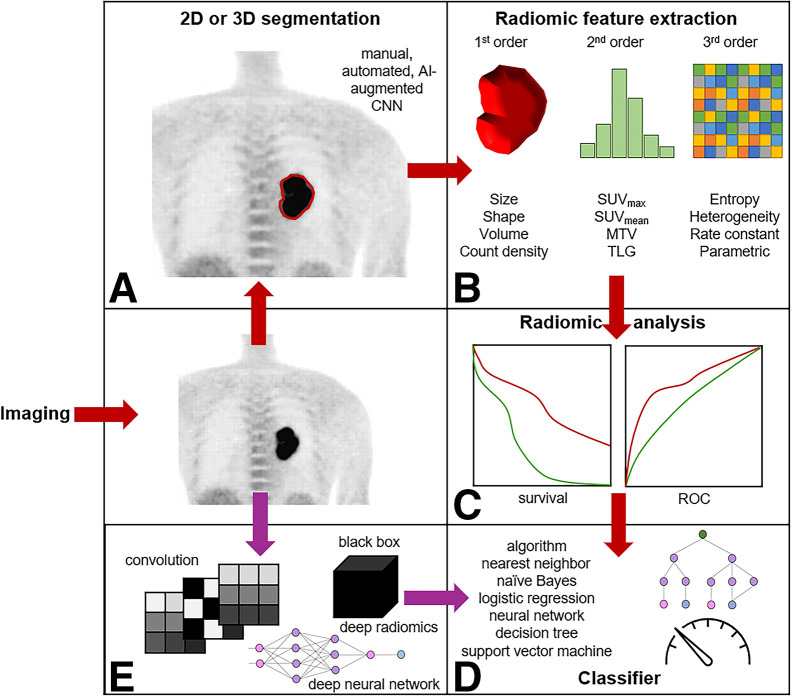
Schematic representation of AI workflow for first-, second-, third-, and fourth-order radiomic features and signatures. Adapted with permission from (49). MTV = metabolic tumor volume; ROC = receiver operating characteristic; TLG = total lesion glycolysis.

**FIGURE 3.**
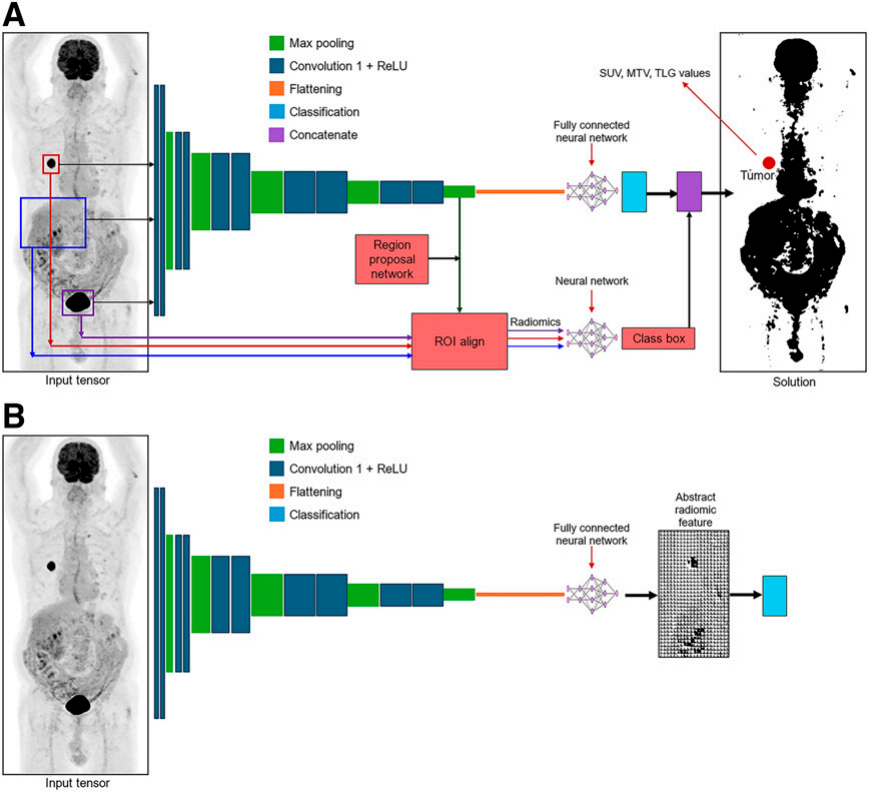
Schematic representation of CNN detection, segmentation, and classification, with lower-order radiomic feature extraction (A). Schematic representation of CNN approach to classification from abstract fourth-order deep radiomic features or signatures (B). Image modified and reprinted with permission from (50). MTV = metabolic tumor volume; ReLU = rectified linear unit; ROI = region of interest; TLG = total lesion glycolysis.

Like theranostics, radiomics is a long-standing principle in nuclear medicine that has been enhanced by recent advances in technology. Radiomic feature extraction is a common application of AI, with handcrafted segmentation giving way to AI-based approaches to manage the increasing complexity and information density of hybrid imaging with SPECT/CT, PET/CT, and PET/MRI. Beyond segmentation, AI can support extraction of higher dimensional features (e.g., texture) from images and abstract features from deep layers of the CNN (48–50). As a result, AI has several roles in radiomic feature extraction (Fig. 2): rapid segmentation of tissues on high density image datasets (Fig. 2A); eliminate interoperator and intraoperator variability in segmentation (Fig. 2A), extraction of first-order (geometry); second-order (intensity) and third-order (texture and wavelet transforms) radiomic features (Fig. 2B); analysis of first-, second-, and third-order radiomic features (e.g., receiver-operator-characteristic analysis, survival) (Fig. 2C); classification of segmented tissues (Fig. 3A) and ML classification based on radiomic feature analysis (e.g., predict response to therapy) (Fig. 2D), and CNN-based extraction of fourth-order (abstract) radiomic signatures (Fig. 3B) for classification (Fig. 2E).

First- and second-order radiomic features are well entrenched in conventional analysis and reporting in nuclear medicine. Certainly, the long-standing role of SUVs and the value of newer parametric variables to PET imaging in predicting response to therapy are crucial to theranostics. For example, DL segmentation can reliably determine metabolic tumor volume and total lesion glycolysis, while reducing the time required to a fraction of that for manual approaches (51,52), DL can localize and classify suspicious lesions (53) and is effective for autosegmentation of metastatic deposits (54). ^177^Lu-PSMA SPECT for radiation dosimetry using SPECT/CT and DL for accurate and fast renal segmentation has significant value in theranostics for monitoring potential renal toxicity (55). Since third- and fourth-order features are more novel, a higher degree of interrogation is required to ensure validity and generalizability, as these are potentially shackled by the lack of transparency of DL algorithms. Nonetheless, fourth-order radiomic signatures can predict patient survival (56), distinguish pathologies or cancer subtypes (57), and predict nodal and distant spread (58). Importantly in theranostics, abstract features extracted from the deep layers of CNNs (i.e., deep radiomics) can predict the response to therapy and radiation dosimetry, which improves patient outcomes (59–61).

A U-Net CNN architecture was trained on ^68^Ga-PSMA PET images and used to accurately delineate gross tumor volume in intraprostatic disease (8). In the validation phase for ^68^Ga-PSMA, there was no statistically significant difference in either sensitivity or specificity of the CNN compared with either expert handcrafted or histologic reference values (8). There was, however, greater variability in performance for ^18^F-PSMA-1007 data. A more successful U-Net CNN was trained on ^18^F-PSMA-1007 data for gross tumor volume delineation with good generalizability across institutions (11). This is important because there are barriers to creating larger multicenter and multinational databases for CNN training.

Textual radiomics of heterogeneity in ^68^Ga-PSMA were shown to be predictive of prostate-specific antigen levels before and after therapy, with ^177^Lu serving as a proxy for predicting responders and nonresponders (31). Specifically, normalized gray-level cooccurrence matrix entropy and homogeneity had negative and positive correlation, respectively, although the areas under the curve for receiver-operating-characteristic analysis were only 0.695 and 0.683, respectively (31). Another study used AI-generated radiomics with ^68^Ga PET/CT to predict overall survival in response to ^177^Lu therapy and reported kurtosis and SUV_min_ as the highest correlated radiomic features (10). Biochemical recurrence in prostate cancer was also predicted (area under the curve, 0.83) using AI-generated PET/MRI radiomic features (32). Extensive details on the AI-driven radiomic use of DOTATATE and PSMA theranostics for predicting response, restaging, and dose prediction have been reported by Yazdani et al. (62) A valuable advantage of radiomic feature extraction and ML-based classification is improved accuracy in the development and validation of nomograms (63).

AI is a powerful tool for rapid and accurate delineation of tumor volumes. This is particularly important in theranostics, where tumor volumes may be numerous and imaged on multiple modalities and over numerous sessions, generating high data density. Handcrafted regions of interest or volumes of interest are time-consuming and introduce interoperator and intraoperator variability. Accurate extraction of radiomic features and signatures can be enhanced by DL. There is a wide array of radiomic features that varies by radiopharmaceutical (48,62,64,65), imaging modality (SPECT/CT vs. PET/CT vs. PET/MRI), and DL algorithm, with novel radiomics emerging regularly (Table 1).

**TABLE 1.** Truncated Overview of Radiomic Features and Signatures Extracted from PET Images in Theranostics (48,62,64,65)

Order and features	Examples	Role of AI
First order: geometry		AI-augmented or CNN-based segmentation
Shape	Variance, homogeneity, surface area, volume, sphericity	
Count density	Counts per pixel/voxel	
Size	Regularity, variance	
Second order: intensity		AI-augmented or CNN-based segmentation and ML classification
Texture spectrum	Max spectrum, symmetry	
Intensity histogram	SUV, GTV, MTV, TLG	
Third order: texture		AI-augmented or CNN-based segmentation and ML classification
GLCM and NGLCM	Contrast, entropy, homogeneity, correlation, dissimilarity	
GLRLM	Variability, intensity, entropy, homogeneity	
GLSZM and GLDZM	Homogeneity, 2D, 3D, pixel fraction	
NGTDM and NGLDM	Coarseness, contrast, busyness, complexity, strength	
Gradient	Absolute, mean, variance, skewness, kurtosis	
Third order: wavelet transforms		AI augmented or CNN-based segmentation and ML classification
Fourier	Frequency features	
Haar	Frequency features	
Gabor	Spatial features	
Gaussian	Spatial features	
Laplacian	Spatial features	
Fourth order: abstract		
High dimensionality from deep layers of neural network	Abstract features	CNN input tensor, DL feature identification, DL classifier

GTV = gross tumor volume; MTV = metabolic tumor volume; TLG = total lesion glycolysis.

## AI AND RADIATION DOSIMETRY

Perhaps the most important application of AI in theranostics is in radiation dosimetry. There are several important roles to discuss. First, AI can be used to augment segmentation across serial and multimodality images to increase accuracy and reduce the time that would be required for handcrafted segmentation. Second, AI tools can help determine radiation dosimetry from serial posttherapy images (e.g., SPECT images of ^177^Lu). Third, AI tools can be used to predict radiation dosimetry using diagnostic images before therapy is administered. Optimal therapeutic doses are associated with a trade-off between tumor dose burden (efficacy of outcomes) and nontarget tissue dose burden (adverse effects and toxicity) that results in individualized patient doses (9). Unlike the individual dose plans of external beam radiotherapy, theranostics tends to adopt a standardized-dose approach (9,34,66). Serial imaging posttherapy using the γ-emissions of the therapeutic radionuclide (e.g., ^177^Lu, ^188^Re) can accurately predict either suboptimal tumor dose burden or nontarget tissue toxicity to allow adjustment of the dose for subsequent cycles. For example, a good tumor dose burden with high renal dose burden may result in a downward adjustment in the subsequent cycle to minimize renal toxicity, while a low tumor dose burden with minimal nontarget tissue dose burden may result in an upward adjustment to improve treatment efficacy (67). One approach compared voxel-based S value U-Net DL dose maps against Monte Carlo simulations for ^177^Lu-DOTATATE SPECT/CT, with mean absolute errors of 1.0% for the kidneys and 3.2% for tumors for the DL approach (68).

Precision medicine in theranostics might demand prediction of radiation dosimetry before the administration of therapy doses. Current approaches rely on insights from physiologically based pharmacokinetics (PBPK), MIRD-based organ dosimetry estimates, and Monti Carlo approaches to model spatiotemporal behavior of the therapeutic probe (9,42,66,69). These models are computationally intensive, with variable accuracy for nonhomogeneous distributions typical of humans and, therefore, have limited application, although they can be enhanced by ML algorithms. AI provides a variety of tools to enhance predictive dosimetry and move toward precision theranostics, as the standardized-dose approach risks undertreatment of tumors or overtreatment of nontarget tissues (70).

A wide array of CNN-based approaches have been adopted for radiation dosimetry prediction. Combined PET images with MIRD-based voxel S values were used to produce an activity map for input into a residual network–style CNN architecture to produce a predictive dose map with a reported 2.6% error from a Monte Carlo–simulated grounded truth (71). A similar comparison to a Monte Carlo–simulated grounded truth used a U-Net CNN architecture to produce a dose rate map (deep-dose) with a 2.5% error (72). Other approaches have included combining PET and CT inputs into a CNN with MIRD S values and empiric mode decomposition (73) and a U-Net CNN trained against Monte Carlo–simulated dose-rate maps (74). Considering the horizon of theranostics, ML has been used to optimize radioligand cocktails for therapy (75). Therapeutic effects may benefit from a cocktail of multiple therapeutic radioligands, but this requires more careful consideration of dosimetry and outcomes. In combinations of 2, 3, and 4 different ^211^At-labeled breast cancer–targeted antibodies, ML produced therapeutic effects with lower cumulative doses (radioactive disintegrations), particularly with 3 and 4 radioligand combinations over single radioligands or the combination of 2 radioligands (75).

In patients with prostate cancer, both the diagnostic images and patient information were inputted into a discriminator neural network architecture to predict spatiotemporal distribution of the therapy probe and the resulting dosimetry (9). The algorithm was trained against actual dosimetry determined from serial posttherapy ^177^Lu images and compared with PBPK approaches. The mean absolute percentage error was lowest for the AI algorithm (15.8% for the kidneys, 25.5% for the liver, 32.1% for the spleen, and 23.8% for the salivary glands) and increased significantly (*P* < 0.01) for the PBPK approach (29.6% for the kidneys, 139.1% for the liver, 54.1% for the spleen, and 67.0% for the salivary glands) (9). A novel approach in ^177^Lu-DOTATATE theranostics used a transformer-based DL model with voxel-based dosimetry trained on ^68^Ga-DOTATATE PET images against posttherapy SPECT/CT images, using Monte Carlo–generated dose maps as the grounded truth (66). The basic architecture was the U-Net CNN, with a transformer integrated into the architecture. Analysis was performed on a voxelwise and organwise basis, with error rates of 5.3% and 1.2%, respectively (66).

**FIGURE 4.**
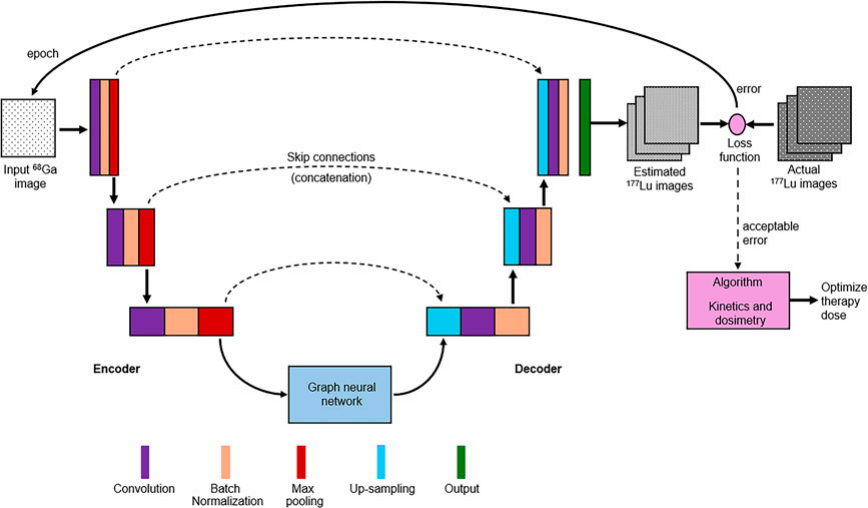
Schematic representation of encoder–decoder graph neural network in U-Net architecture to predict ^177^Lu dosimetry based on ^68^Ga PET. Adapted with permission (33).

Digital twins create a digital replica of an actual object (Fig. 4) using the same input data, while allowing updates to the digital twin in response to new information (33,76). A simple digital twin model of human biology can be used to estimate organ doses based on changes to variables. A more complex digital twin model accommodates pharmacokinetics using compartment modeling (33). Advances in ML and DL have created the opportunity for the deployment of digital twins in radiation dosimetry (33). The one-size-fits-all approach to therapy doses is an area that could benefit from the nexus of AI and digital twins. PBPK modeling adopts several mathematic models, each with its own set of limitations (69). Calculations are model-based and include assumptions based on historical data, standardization, and normal physiology. Current approaches to radiation dosimetry based on therapy dose kinetics allows only reactive rather than proactive action. As discussed previously, the diagnostic distribution does not accurately predict therapy biodistribution across the wider therapeutic window. Incorporating AI and PBPK digital twins into predictive dosimetry could drive precision theranostics. Fundamentally, a CNN (U-Net) and DL could be used to predict ^177^Lu dosimetry from a ^68^Ga scan by training the algorithm to predict dosimetry from ^68^Ga PET against serial ^177^Lu scans as the grounded truth (Fig. 4), with an error rate of approximately 2% (72,73,77).

Generative adversarial networks (GANs) use a discriminator neural network in tandem with a generator neural network to produce generated (fake) images that are fed into the discriminator network for classification, thereby boosting the training dataset (1,33,78,79). GAN-based DL CT dosimetry has been developed to calculate organ doses from the original and generated images in real time to personalize CT dosimetry (80). Since digital twins are digital models of actual objects, GANs could produce accurate digital twins that could be used for ^177^Lu radiation dosimetry. The digital twins could model dosimetry based on variations to the therapeutic dose before the administration of the therapeutic dose, which would afford the opportunity to optimize the actual patient dose. That is, the digital twins can be modeled on tumor, kidney, and other critical organ PBPK, and variations to therapy doses will produce predicted dosimetry to allow the optimal combination (dose, tumor dose, nontarget tissue dose) to be selected and then delivered to the patient.

## AI, DRUG DEVELOPMENT, AND PRECLINICAL IMAGING

**FIGURE 5.**
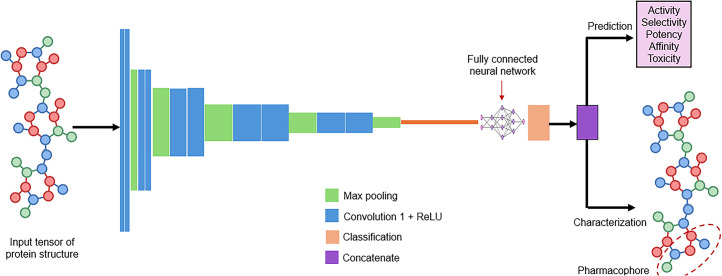
Schematic representation of CNN encoding protein structure to identify potentially important pharmacophores and chelation suitability, and pharmacodynamic and pharmacokinetic properties. ReLU = rectified linear unit.

There is a long history of the use of AI methods in drug design (35). The role of AI in drug development is to improve the process timelines, improve productivity, reduce cost, reduce failure rates, and improve sustainability (35,70,81). ML approaches have enhanced drug development with better data interrogation with nonlinear modeling (82). It is sensible to translate these AI developments from drug development to radioligand development. There are 3 general approaches to AI augmentation of radioligand development. Ligand-based approaches focus on pharmacophore modeling for receptor selectivity, structure-based approaches focus on pharmacodynamics and binding affinity, and pharmacokinetics-based approaches focus on absorption, distribution, metabolism, elimination, and toxicity (35,83). AI, particularly DL, has significant potential for pharmacophore modeling to understand chemical patterns, identify models of greatest potential, and shorten development time (35,84). DL can predict the structure–activity relationship of molecules, which may identify new radioligand candidates (85,86). Although computer-aided, ML and in silico techniques have been reported for radioligand development (70,83,87), the benefits of DL in the drug development pipeline are yet to be fully realized in radioligand development (35), but the pursuit of novel theranostic pairs will likely benefit from adopting these established AI approaches (Fig. 5). Indeed, beyond radioligand development, ML and DL may provide insights into novel radionuclides suitable for diagnostic or therapeutic pairs or optimal chelation. Nonetheless, ligand-based modeling with the aid of virtual screening was used to identify candidates for PET imaging of tauopathies (88). The value of AI is in the rapid evaluation of large quantities of structural and biologic data. A novel approach would be to use the GAN to design new biomolecules for use in theranostics by using existing useful and unsuccessful radioligands as input into the discriminator neural network (85,89).

Preclinical imaging addresses several challenges associated with the size of the organs being imaged and the instrumentation designed to perform the imaging. Limitations in spatial resolution produce limitations in quantitation accuracy. Limitations in sensitivity produce noisy images. Multipinhole collimators produce complex datasets and distorted images that, combined with more challenging organ segmentation, make coregistration more difficult. The previously discussed general benefits of and opportunities afforded by DL architecture could enhance spatial resolution of and denoise preclinical images, and enhance segmentation, reconstruction, and coregistration. More specifically, CNN U-Net architecture can denoise low-count SPECT and PET images (35). It is important to recognize that this approach neutralizes motivation to increase the radiation dose to individual mice (improving animal care and reducing costs), but CNN denoising models do not improve count statistics. Animal motion seen in longer imaging times could be addressed by shorter imaging times with denoising algorithms to address lower counts. To translate human PET system spatial resolution to preclinical imaging in mice, a 10-fold improvement in spatial resolution is required (35).

Preclinical imaging is an essential part of the development of theranostic pairs; however, there are several ethical, political, and social challenges that apply pressure to reduce animal use, refine protocols, and, where possible, replace animal use (36). The homogeneity of mice (relative to humans) is conducive to “whole-mouse” digital twins. DL-powered digital twins could reimagine the preclinical ecosystem in theranostics. Although interesting, digital twins will not displace the need for preclinical animal imaging. Instead, digital twins could provide a narrow range of insights that reduce the use of animals for those specific questions (e.g., renal absorbed dose for different administered doses).

## GENERATIVE AI

Foundation models, such as LLMs, diffusion models, and VLMs, are pretrained with significant versatility to be applied to tasks for which they were not originally designed (40). Some potential applications include scenarios where data are limited (40), which could provide benefit to theranostic data when barriers prevent pooling of data from multiple centers. Unfortunately, foundation models have inherent risks of overgeneralization and privacy breaches and tend to not adapt well to highly specialized tasks (40). Federated learning remains a potential solution to privacy issues. Some of the emerging applications of foundation models include improved management and processing complex, serial, or sequential data that lacks rigorous annotation; few- and zero-shot learning capability, particularly useful in scarce or highly heterogenous data; accessibility and understandability capabilities for the dense knowledge intensity ecosystem of theranostics to improve information dissemination and decision-making; prediction of sequences and patterns in complex biologic data; rapid and concise synthesis of large and varied information sources; multimodality integration of diverse data to inform predictive analytics; and a second-reader system for data-driven error and ambiguity minimization (40).

Some examples include the adaptation of bidirectional encoder representations from transformers (BERT) for BioBERT, Med-BERT, and ClinicalBERT, which add medical texts and scientific publications to their corpora (90–92). Similarly, LLMBiomedicine (93) and ClinicalGPT (94) use the GPT backbone with biomedical texts, and ChatDoctor is an LLM Meta-AI–based patient–physician conversational chatbot (95). These general medical applications could be adapted or modeled for theranostic-specific versions to provide theranostic information for patients and clinicians and provide conversation chatbots to better support theranostic patients with care before and after therapy.

**FIGURE 6.**
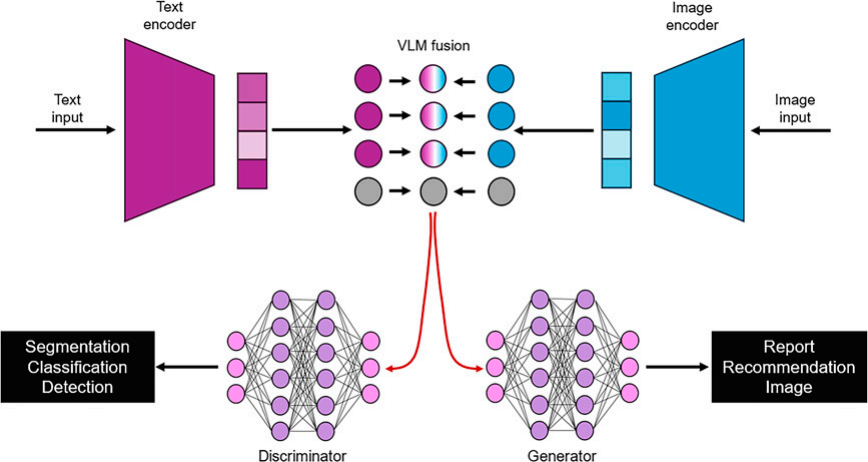
VLM with fusion of text and image inputs to create traditional outputs from discriminator network in tandem with generated report, recommendation, or treatment outcome prediction and generated images.

VLMs are multimodality transformers (Fig. 6) that are trained using contrastive learning to perform tasks that connect text features with image features (47). VLMs can be used to extract higher dimensional features from images integrated with semantic information to provide deeper insights into diagnosis, classification, and treatment planning (47). Although VLMs are only recently emerging in medical image analysis, they have several potential advantages for use in the theranostics space. First, current AI methods for segmentation and classification rely on large training sets of diverse data. VLMs have the potential for few- and zero-shot learning, meaning that they could perform classification, segmentation, and detection tasks on data on which it has not been trained (47). Second, VLMs support knowledge transfer between modalities (47), which could be beneficial in improving the predictive capability of AI by converting transdisciplinary semantic and visual data into a common language. This approach also integrates the granular detail of semantic data with the abstract features of images. Third, VLMs may provide a means for better predictive radiation dosimetry by incorporating both the high- dimensional insights of AI-based image analysis with the clinical expertise detailed in the semantic inputs, provided the semantic report was deliberate in adding this information. Among various general biomedical VLMs, CheXZero uses a contrastive language–image pretraining (CLIP)–based image encoder and text encoder for zero-shot learning to accurately identify pathology on chest radiograph (96). EchoCLIP uses a ConvNeXt image encoder and CLIP text encoder trained on more than 1 million echocardiograms to enhance interpretation (97). Although neither application is specific to theranostics, they do provide insight into developments that could, and perhaps will in the short-term future, be adapted to theranostic applications such as staging, restaging, predicting response to therapy, and radiation dosimetry.

## AI AND SUSTAINABILITY IN THERANOSTICS

Sustainability is often associated with lowering the carbon footprint, improving environmental impact, and implementing “greener” practices. Sustainability also relates to practices that ensure the durability and longevity of theranostic services to communities. For theranostics, sustainability requires balancing consumption with resource availability to avoid depletion exceeding regeneration (98). The role of AI in theranostics sustainability is multidimensional. Both ML and DL applications have been built to create workflow efficiencies that create sustainability of the workforce (e.g., reduce burnout) and enhance patient-centered care (37). Furthermore, AI can be used to improve workplace diversity and inclusivity (38) and address health care inequities across communities (99). Emerging generative AI applications aim to enhance workforce training to produce efficiencies and cost reductions and enrich patient education (37). Theranostics is often associated with expensive products and services, which tend to reflect, in part, high research and development costs. The use of DL approaches for both drug development and in silico alternatives to preclinical imaging provides sustainability in economic and social domains (37). The previously discussed dose-reduction strategies supported by DL resolution enhancement and denoising could produce sustainability in theranostics through a better radiation safety profile, better use of scarce resources (e.g., ^177^Lu and ^68^Ga), and cost reductions. Likewise, DL-based radiation dosimetry approaches not only optimize multiple cycles of therapy doses but also improve outcomes to drive a more sustainable theranostic ecosystem. DL-driven digital twins could reduce wastage, costs, and time associated with theranostic research and development, contributing to improved sustainability (33). For example, digital twins could circumvent preclinical imaging bottlenecks using in silico approaches.

Research and development costs in radioligand development can be extraordinarily high due to a 80%–90% failure rate at the clinical phase (35,81). There are potential opportunities for AI to augment radioligand development by reducing development time, investment in unsuccessful products, and costs and by improving sustainability in the sector (35,81,82). AI has been used in drug discovery and development across the product cycle from drug discovery to product management, including identifying therapeutic targets and target protein structure prediction, drug or target protein interaction prediction and modeling, de novo drug design, predicting bioactivity and toxicity, predicting and designing pharmacologic and biospecific properties, identifying and optimizing excipients, optimizing the development process, monitoring compliance, predicting new or novel therapeutic targets, and powering in silico methods of drug evaluation, including AI-based digital twins. (81,100,101). These roles of AI are equally relevant to improving the sustainability of radioligand development in theranostics (35,83,102).

Despite the potential value of AI in theranostics, ethical considerations remain (103,104). Given the ongoing ethical debate around privacy, confidentiality, and data ownership, federated learning provides a method for training ML and DL algorithms on rich and diverse data from multiple decentralized sources without the need to transfer or share local data or information (39–42). Federated learning is a distributed learning approach that is crucial for sustainable AI in theranostics because, without these developments, training and validation data will lack diversity, be too small to be useful, and will prevent external validity and generalizability. Although federated learning is a key component of AI-based radiation dosimetry developments, there are also important applications of federated learning for the use of foundation models in generative AI (text-to-text and text-to-image applications) (40). This could broaden the applications of generative AI in clinical and research practice and expand the capabilities of foundation models by exploiting their versatility.

**FIGURE 7.**
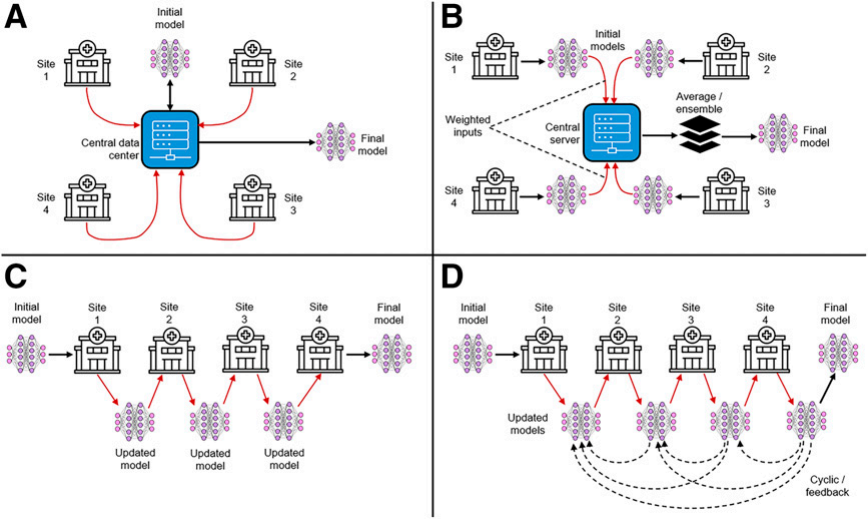
**(**A) Centralized data-sharing model that lacks privacy. (B) Weighted federated average approach (ensemble method). (C) Federated transfer learning (single pass and sequential). (D) Cyclic (recurrent) federated transfer learning passed though institutions multiple times.

One approach to federated learning uses weighted federated averaging (Fig. 7). This involves training the model on local datasets at each site and then pooling (averaging) the multiple trained models for implementation more universally (41). This process is iterative in nature, with ongoing learning from new data. Conversely, federated transfer learning (Fig. 7) uses a central model trained on decentralized data and then optimized for implementation at each site (41).

## CONCLUSION

Richard Baum is often credited with the theranostics maxim, “We see what we treat, and we treat what we see” (105). Standardized-dose strategies for therapy defy this maxim. The role of AI in theranostics is a powerful step toward realizing this maxim and moving toward precision medicine and precision theranostics. AI-augmented segmentation and radiomic feature extraction enhance “what we see.” DL approaches to radiomic feature analysis and predictive dosimetry enhance “what we treat.” At the same time, AI-augmented radioligand development allows novel or more targeted theranostics, sharpening vision and treatment. The application of AI in sustainability is of growing importance, and the emergence of generative AI promises exciting opportunities. Nonetheless, there remain limitations and challenges associated with algorithm generalizability, transparency, accountability, diversity, and privacy. AI has the potential to revolutionize the theranostics ecosystem and move toward precision medicine.

## DISCLOSURE

No potential conflict of interest relevant to this article was reported.
